# Supplementary material for: Topology-Driven Discovery of Transmembrane Protein S-Palmitoylation
Source: bioRxiv. 2024 Sep 8:2024.09.08.611865. Preprint. [Version 1] doi: 10.1101/2024.09.08.611865 (PMC11398512; doi:10.1101/2024.09.08.611865)
Supplement: Supplement 1 [file media-1.pdf]

SUPPORTING INFORMATION

Topology-Driven Discovery of Transmembrane Protein *S*-Palmitoylation

Michael T. Forrester[1,]* Jacob R. Egol[2], Sinan Ozbay[3], Rohit Singh[2,3] and Purushothama Rao Tata[1,2,4]

[1]Division of Pulmonary, Allergy and Critical Care Medicine

[2]Department of Cell Biology

[3]Department of Biostatistics and Bioinformatics

[4]Duke Regeneration Center

Duke University School of Medicine, Durham, NC 27710

TABLE OF CONTENTS

Supplementary methods 2

Figure S1. Summary statistics of the training dataset 5

Figure S2. Hydrophobicity related features 6

Figure S3. Amino-acid specific features 7

Figure S4. Sequence logos based on *S*-palmitoyl status 8

Figure S5. Feature importance plot for TopoPalmTree. 9

Supplementary Table Descriptions 10

Table S1. TopoPalmTree applied to viral *S*-palmitoyl protein dataset (xls)

Table S2. TopoPalmTree applied to the mouse transmembrane proteome (xls)

SUPPLEMENTARY METHODS

Synthesis of pyridyl disulfide sepharose for Acyl-RAC – All reactions are conducted in PDS buffer (100 mM HEPES + 2 mM EDTA pH 7.8) unless otherwise indicated. A 3 mL slurry of NHS-Sepharose was washed three times with PDS buffer in a 10 mL filter column, followed by addition of 100 mM cystamine in PDS buffer to fill the 10 ml column. The tube is rotated for 4 hours at room temperature, washed at least 4 times with PDS buffer, washed 4 times with PDS buffer containing 20 mM DTT, then washed another 4 times with PDS without DTT. The slurry is washed 4 times with MeOH, 100 mM 2-pyridyl disulfide (aka Aldrithiol-2) in MeOH is added to fill the column and the reaction is rotated at room temperature for 15 minutes. The slurry is washed twice with 100 mM 2-pyridyl disulfide in MeOH then three times with MeOH, then finally isopropanol. The slurry is stored at 50 % density (e.g. 2 ml of sepharose in 4 ml of isopropanol) in a sterile 15 ml conical tube at 4°C.

Resource Table

REAGENT or RESOURCE	SOURCE	IDENTIFIER
**Antibodies (dilution)**		
Flag M2 monoclonal Ab (1:4000)	Sigma	F3165
Flag monoclonal Ab (1:4000)	Proteintech	66008-4-Ig
Vamp5 polyclonal Ab (1:1000)	Proteintech	11822-1-AP
Aqp5 polyclonal Ab (1:1000)	Cell Signaling Technologies	59558
**Reagents for synthesis of thiopropyl sepharose (TPS)**		
Sepharose-NHS	Sigma	H8280
Cystamine-HCl	Sigma	C121509
Alrithiol-2 (2-PDS)	Sigma	143049
Pierce Centrifuge Columns, 10 mL	Pierce	89898
**Enzymes**		
Q5 Polymerase	New England Biolabs	M0491
T4 Ligase	New England Biolabs	M0202
Kinase / Ligase / Dpn1 (KLD Mix)	New England Biolabs	M0554S
AgeI-HF	New England Biolabs	R3552
EcoRI-HF	New England Biolabs	R3101
NotI-HF	New England Biolabs	R3189

**Kits for cloning**		
RNeasy	Qiagen	74104
Qiaex II Gel Extraction Kit	Qiagen	20021
SuperScript III First Strand Kit	Thermo	18080-400
**Cell Lines and Culture Reagents**		
HEK293	Duke Cell Culture Facility	N/A
High Glucose DMEM	Gibco / Thermo Fisher	11965092
FBS	Cytiva Hyclone	SH30071
Pen-Strep		
Polyethylenimine (PEI)	Polysciences	23966
Trypsin-EDTA (0.25%)	Gibco / Thermo Fisher	25200056
**Mammalian expression vector**		
pCMV-EGFP	Addgene	11153
**Oligonucleotides for cloning and annealed oligo ligation**		
See DNA oligos table below		
**Chemicals / General Reagents**		
2-Bromohexadecanoic acid (2-bromopalmitate)	Sigma	238422
N-Hydroxysuccinimidyl-Sepharose™ 4 Fast Flow	Sigma	H8280
2-Pyridyl disulfide (Aldrithiol-2)	Sigma	143049
Cystamine dihydrochloride	Sigma	C121509

DNA oligos used for PCR and cloning – Sequences are all for murine genes. Fw = forward. Rv = reverse. Tm = melting temperature. Ta = annealing temperature used for PCR. For all reactions, Q5 polymerase was used except for Vamp5 cloning into pCMV-EGFP which was performed with Dream Taq.

Primer Name	Sequence (5’ -> 3’)	Tm / Ta (C)
**Cloning into pCMV-EGFP**		
Vamp5_EcoRI_Fwd	ATTAGAATTCACCATGGCAGGGAAAGAACTGAAGCAATGCCAGC	70 / 66

Vamp5_AgeI_Rev	ATTAACCGGTGGTTTACTACTGTCCCCACCACTCGGAAG	71 / 66
Aqp5_EcoRI_Fwd	TATAGAATTCACCATGAAGAAGGAGGTGTGTTCAGTTGC	65 / 68
Aqp5_AgeI_Rev	TAATACCGGTAAGTGTGCCGTCAGCTCG	67 / 68
KdelR2_EcoRI_Fwd	ATTAGAATTCACCATGAACATCTTCCGGCTGACTGGGGACCTG	70 / 72
Kdelr2_AgeI_Rev	TTAAACCGGTGCTGGCAGGCTGAGCTTCTTCCCTTTGAGTAC	73 / 72
**Site Directed Mutagenesis**		
Vamp5_C9S_Fwd	ACTGAAGCAAAGCCAGCAGCAGG	70 / 66
Vamp5_C9S_Rev	TCTTTCCCTGCCATGGTG	65 / 66
Vamp5_C69S_Fwd	AAATATCCGGAGCCGGGTCTACT	67 / 67
Vamp5_C69S_Rev	TCCCAGCGCTTCTGC	66 / 67
Aqp5_C6S_Fwd	GAAGGAGGTGAGCTCAGTTGCCT	64 / 61
Aqp5_C6S_Rev	TTCATGGTGAATTCGAAGC	60 / 61
Aqp5_C145S_Fwd	GCTGGCCCTCAGCATCTTCTCCT	70 / 66
Aqp5_C145S_Rev	TTCATGGTGAATTCGAAGC	65 / 66
KdelR2_T200C_Fwd	CTTGTACATTTGCAAAGTACTCAAAGGGAAGAAG	57 / 58
KdelR2_T200C_Rev	TAGAAGAAGTCGCAGTAG	58 / 58
**Annealed oligos for EGFP removal and flag epitope insertion at Age I / Not I sites**		
EGFP_to_flag_Fwd	CCGGTAGACTACAAAGACGATGACGACAAGTGATCTAGAGC	N/A
EGFP_to_flag_Rev	GGCCGCTCTAGATCACTTGTCGTCATCGTCTTTGTAGTCTA	N/A

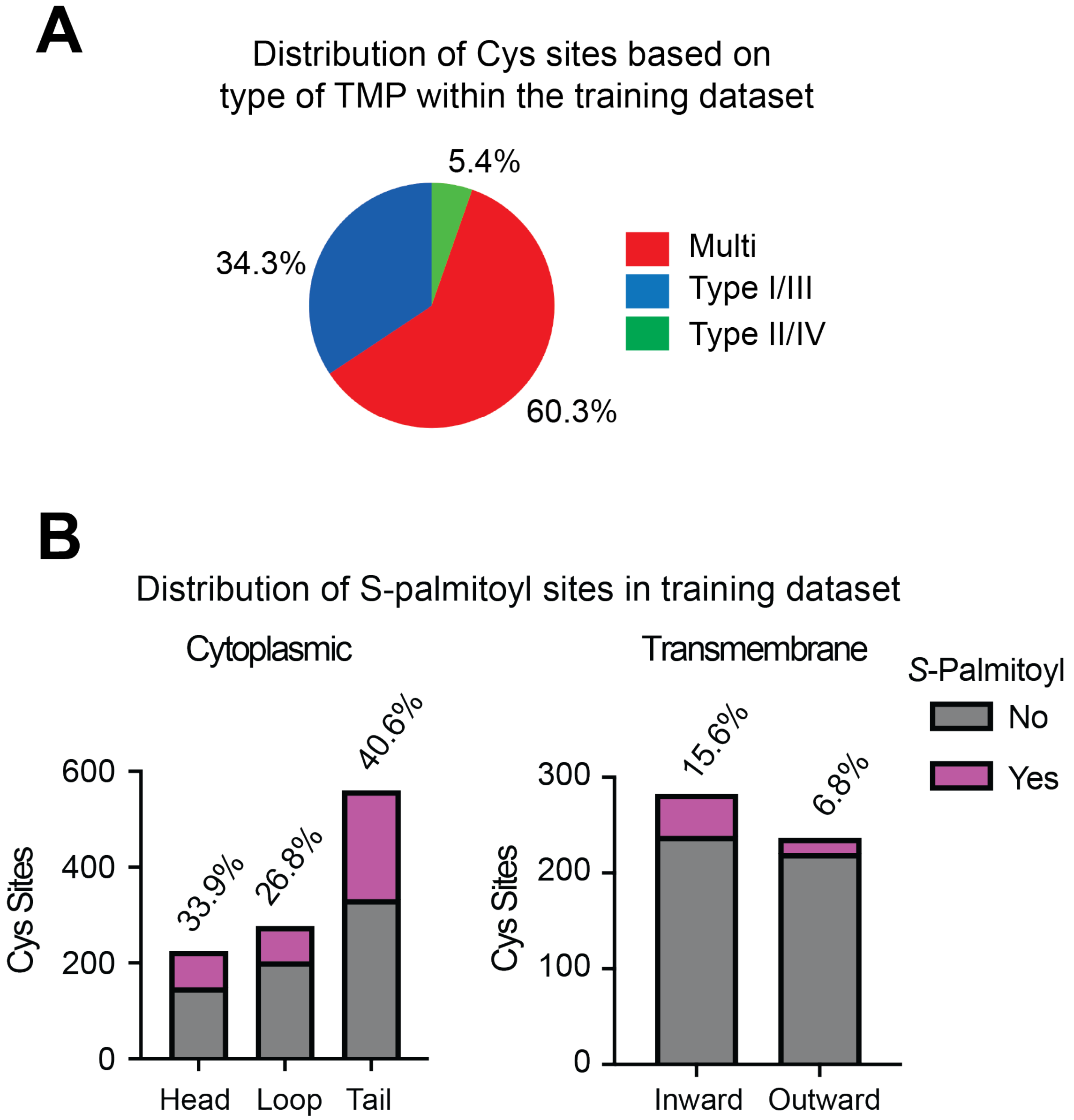

Figure S1. Summary statistics of the training dataset. (A) Types of TMPs represented in the training data. Total number of Cys sites are grouped based on whether they reside on a multi-pass, Type I/III or Type II/IV TMP (based on orientation of the N-termini relative to the lipid bilayer). (B) Distribution of *S*-palmitoyl (magenta) and non-*S*-palmitoyl (gray) Cys sites based on topological location. Shown above each column are the percentages of Cys sites that are *S*-palmitoylated within each type.

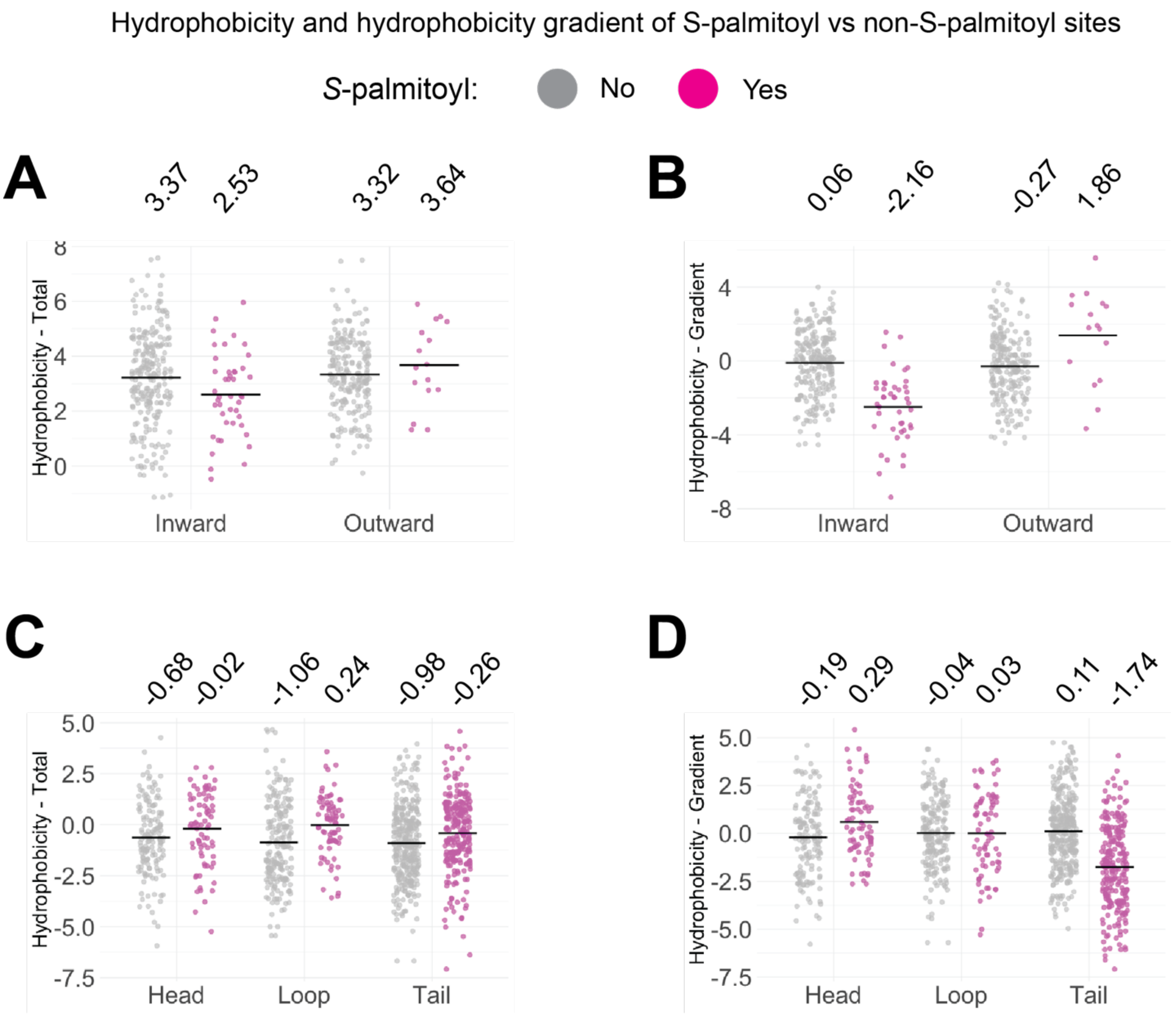

Figure S2. Hydrophobicity and hydrophobicity gradients as examples of the TopoPalmTree feature set. Hydrophobicity values are derived from the window sequence (5 amino acids flanking on each side of the given Cys residue). Total hydrophobicity is calculated for the entire window sequence whereas the hydrophobicity gradient is the difference in hydrophobicity (C-terminal minus N-terminal window). For total hydrophobicity, positive values indicate increased overall hydrophobicity surrounding the Cys residue. For hydrophobicity gradient, more positive values indicate increasing hydrophobicity from the N- to C-terminal direction across the given Cys residue. Shown are (A) total hydrophobicity and (B) hydrophobicity gradient for transmembrane regions, as well as (C) total hydrophobicity and (D) hydrophobicity gradient for cytoplasmic regions. The black bars represent mean values.

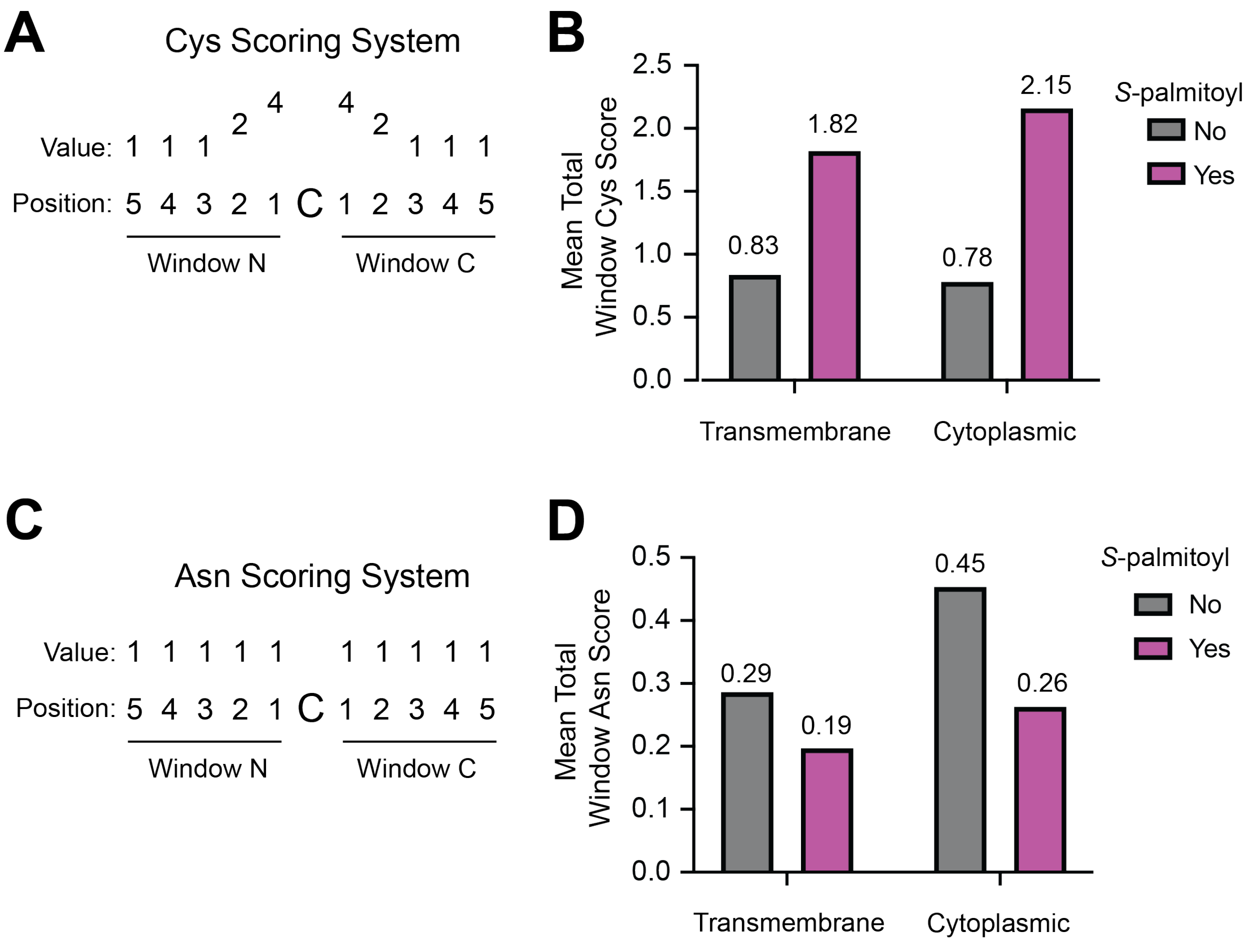

Figure S3. Window Cys score and Asn scores as examples of amino acid-based features within the training dataset. (A) Window sequence scoring system for adjacent Cys residues giving priority to closer Cys residue. Cys residues flanking the Cys of interest (center) are awarded 4 points. Cys residues in the 2nd flanking positions are given 2 points, and then 1 point for any Cys within flanking positions 3 through 5 in either direction. (B) The values are then summed to achieve a total score for the N- and C-terminal windows, with mean values shown based on *S*-palmitoyl status. (C) Asparagine scoring system providing one point for every Asn residue within the N- and C-terminal windows. (D) Mean total Asn scores based on *S*-palmitoyl status.

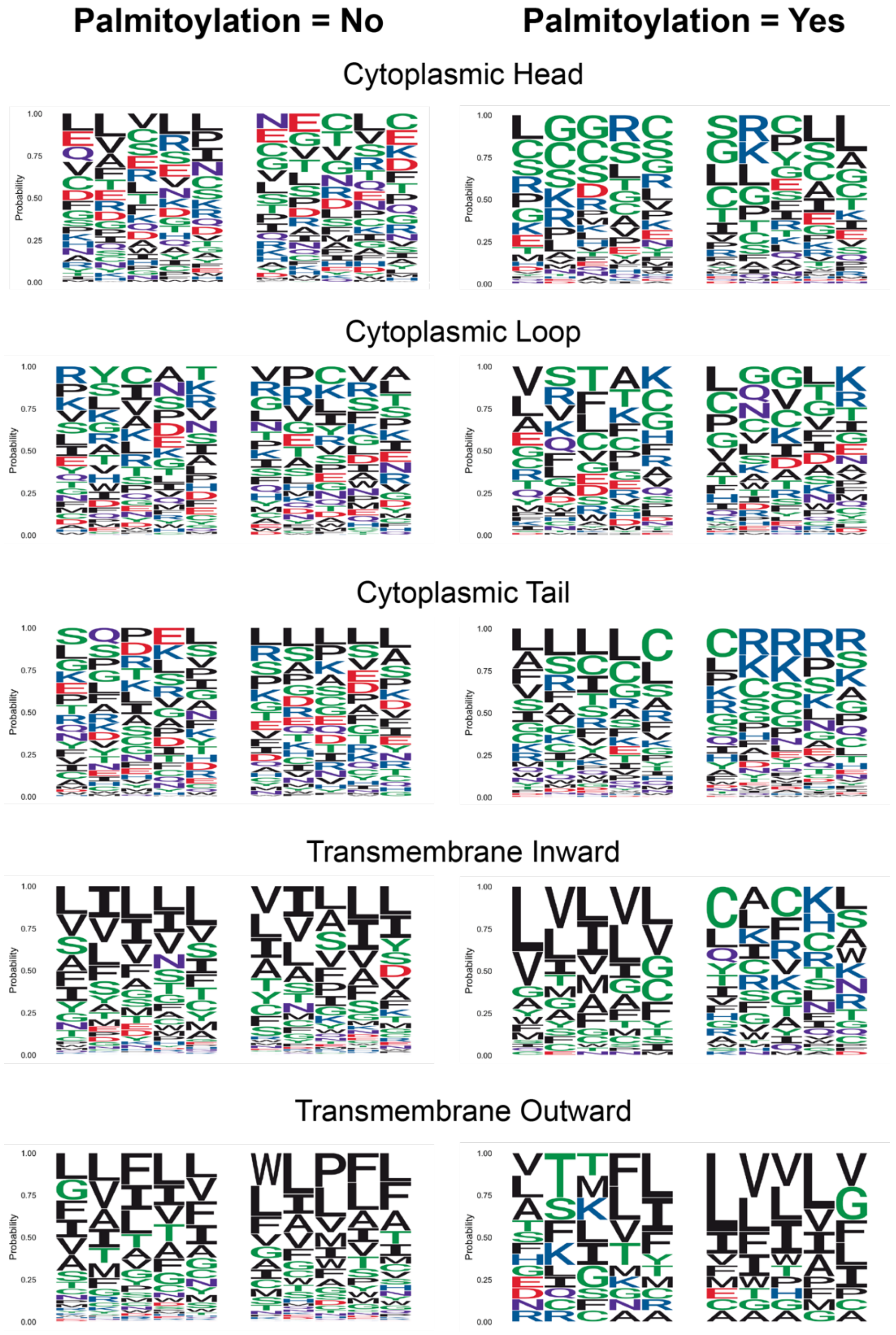

Figure S4. Sequence logos based on *S*-palmitoyl status within the training dataset. Sequence logos were generated with package ggseqlogo. For each logo, the central empty space represents the Cys site of interest with N- and C-terminal flanking residue probabilities on the left and right, respectively. The y-axes represent probability with each position summing to 1. Logos are separated by column for *S*-palmitoyl status and row for topological locations.

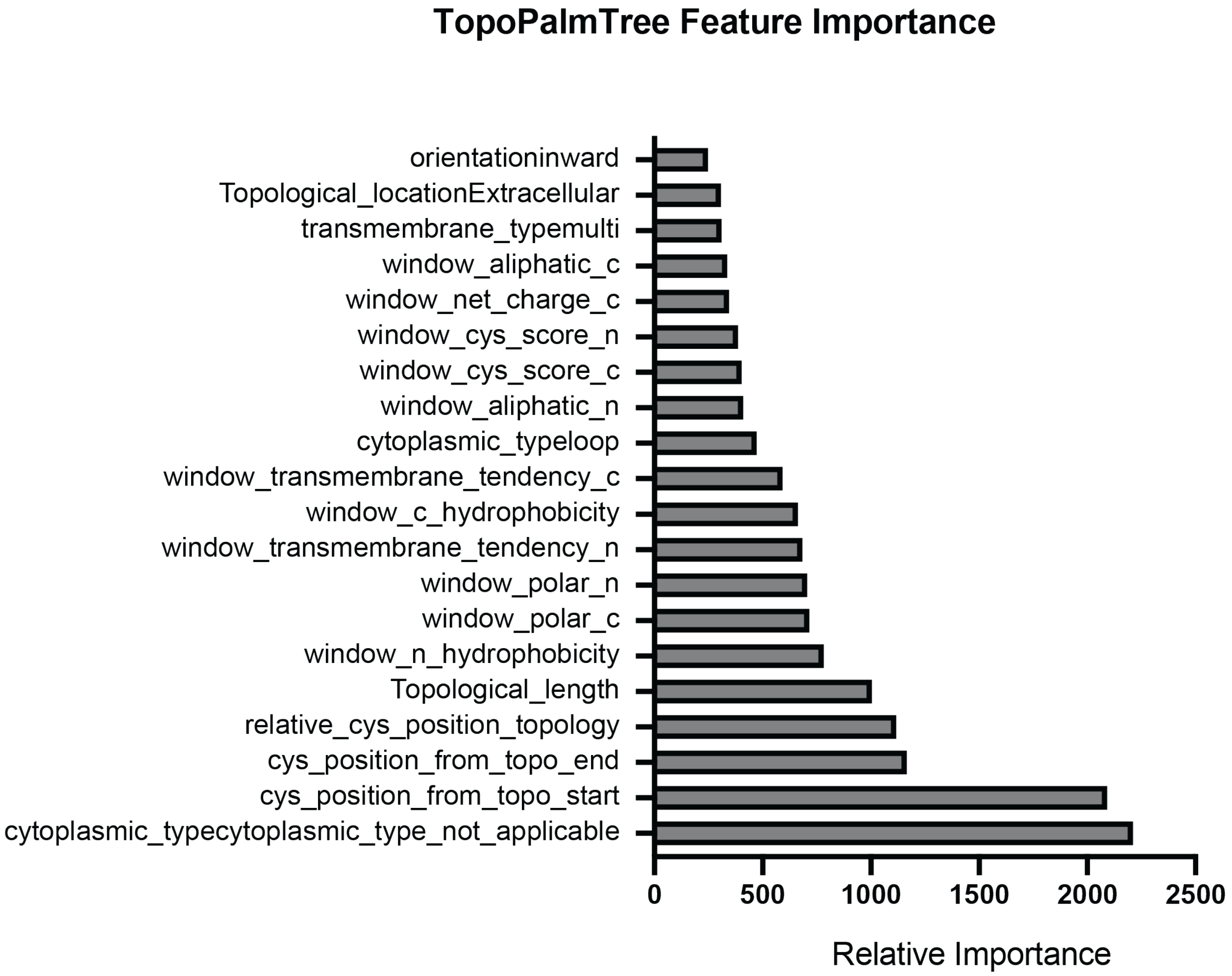

Figure S5. Feature importance plot for TopoPalmTree. The varImp function from the caret package was used to examine feature importance for the top 20 features. Importance is determined by reduction in the Gini index. The highest scoring feature determines whether the Cys site is cytoplasmic. Features ending with _n and _c represent the N- and C-terminal windows, respectively, for the indicated feature.

SUPPLEMENTARY TABLES

Table S1. Results of TopoPalmTree applied to the holdout viral *S*-palmitoyl protein dataset. Class1 probability reflects the TopoPalmTree output score for a positive class (i.e. *S*-palmitoyl) assignment.

Table S2. TopoPalmTree applied to the mouse transmembrane proteome. Class1 probability reflects the TopoPalmTree output score for a positive class (i.e. *S*-palmitoyl) assignment. A total of 49828 Cys residues were amenable to inference by TopoPalmTree. A minority of the Cys residues (5562) were located in rare locations (e.g. signal sequences) not represented in the training dataset and are considered not amenable to inference. Those sites and any other sites not amenable to inference have NA values in the probability column.
